# Predicting Cardiovascular Risk in Patients with Prostate Cancer Receiving Abiraterone or Enzalutamide by Using Machine Learning

**DOI:** 10.3390/cancers17152414

**Published:** 2025-07-22

**Authors:** Dong-Yi Chen, Chun-Chi Chen, Ming-Lung Tsai, Chieh-Yu Chang, Ming-Jer Hsieh, Tien-Hsing Chen, Po-Jung Su, Pao-Hsien Chu, I-Chang Hsieh, See-Tong Pang, Wen-Kuan Huang

**Affiliations:** 1 Division of Cardiology, Department of Internal Medicine, Chang Gung Memorial Hospital at Linkou, Taoyuan 33302, Taiwan; dongyi70@cgmh.org.tw (D.-Y.C.); mr3228@cgmh.org.tw (C.-C.C.); b9402045@cgmh.org.tw (C.-Y.C.); a12390@cgmh.org.tw (M.-J.H.); pchu@cgmh.org.tw (P.-H.C.); v12001@cgmh.org.tw (I.-C.H.); 2 Graduate Institute of Clinical Medical Sciences, College of Medicine, Chang Gung University, Taoyuan 33302, Taiwan; 3 College of Life Sciences and Medicine, National Tsing Hua University, Hsinchu City 30013, Taiwan; 4 College of Medicine, Chang Gung University, Taoyuan 33302, Taiwan; 8902024@cgmh.org.tw (M.-L.T.); skyheart0826@cgmh.org.tw (T.-H.C.); pjsu@cgmh.org.tw (P.-J.S.); 5 Division of Cardiology, New Taipei Municipal TuCheng Hospital, New Taipei City 236017, Taiwan; 6 Division of Cardiology, Department of Internal Medicine, Chang Gung Memorial Hospital at Keelung, Keelung City 20401, Taiwan; 7 Division of Hematology/Oncology, Department of Internal Medicine, Chang Gung Memorial Hospital at Linkou, Taoyuan 33302, Taiwan; 8 Division of Urology, Department of Surgery, Chang Gung Memorial Hospital at Linkou, Taoyuan 33302, Taiwan

**Keywords:** prostate cancer, abiraterone, enzalutamide, major adverse cardiovascular event, machine learning, random survival forest

## Abstract

**Purpose:** The identification of cardiovascular risk factors in metastatic prostate cancer (PCa) patients prior to the initiation of androgen receptor pathway inhibitors (ARPIs) is important yet challenging. **Methods and Results:** A nationwide cohort study was conducted utilizing data from the National Health Insurance Research Database containing the Taiwan Cancer Registry. The study population comprised 4739 PCa patients who received abiraterone or enzalutamide between 1 January 2014, and 28 February 2022. The cohort was divided into a training set (*n* = 3318) and a validation set (*n* = 1421). Machine learning techniques with random survival forest (RSF) model incorporating 16 variables was developed to predict major adverse cardiovascular events (MACEs). Over a mean follow-up period of 2.1 years, MACEs occurred in 10.9% and 11.3% of the training and validation cohorts, respectively. The RSF model identified five key predictive indicators: age < 65 or ≥75 years, heart failure, stroke, hypertension, and myocardial infarction. The model exhibited robust performance, achieving an area under the curve (AUC) of 85.1% in the training set and demonstrating strong external validity with an AUC of 85.5% in the validation cohort. A positive correlation was observed between the number of risk factors and the incidence of MACEs. **Conclusions:** This machine learning approach identified five predictors of MACEs in PCa patients receiving ARPIs. These findings highlight the need for comprehensive cardiovascular risk assessment and vigilant monitoring in this patient population.

## Simple Summary

Prostate cancer patients receiving newer hormone treatments (abiraterone or enzalutamide) face an increased risk of heart problems, yet identifying vulnerable patients beforehand remains challenging. Our study developed a prediction tool to assess cardiovascular risk in these patients using data from over 4700 individuals. Through advanced machine learning techniques, we identified five key risk factors: age (under 65 or over 75), previous heart failure, stroke history, high blood pressure, and previous heart attack. Our model demonstrated excellent predictive accuracy in both testing and validation groups. Importantly, we found that patients with more risk factors experienced significantly higher rates of heart complications. This practical risk assessment tool can help doctors identify high-risk patients before starting treatment, enabling personalized monitoring strategies and potentially improving the safety of these important cancer medications.

## 1. Introduction

The therapeutic landscape for advanced prostate cancer (PCa) has evolved significantly over the past decade. While androgen deprivation therapy (ADT) with gonadotropin-releasing hormone (GnRH) agonists or antagonists remains the cornerstone of treatment, the emergence of the androgen receptor pathway inhibitor (ARPI) has transformed patient care. Two agents in particular—abiraterone acetate and enzalutamide—have demonstrated substantial clinical benefits when combined with ADT. These drugs work through distinct mechanisms; abiraterone inhibits androgen biosynthesis through CYP17 blockade and requires concomitant corticosteroids to prevent mineralocorticoid excess, while enzalutamide acts as a potent androgen receptor antagonist that also prevents nuclear translocation of the receptor [1]. These drugs were approved for metastatic castration-resistant PCa (mCRPC) based on landmark trials showing improved survival in both the pre- and post-chemotherapy settings [2,3,4,5]. The expanding therapeutic scope has led to their widespread adoption, making them the predominant systemic treatment in combination with ADT for advanced PCa.

However, the therapeutic benefits of abiraterone and enzalutamide must be weighed against their potential cardiovascular risks, particularly when combined with ADT in an already vulnerable patient population [6]. Multiple real-world studies have demonstrated increased cardiovascular morbidity with these agents. Abiraterone has consistently shown a higher cardiovascular risk profile, with studies reporting a 31–77% increased risk of major cardiovascular events compared to ADT alone, likely related to its mineralocorticoid effects despite corticosteroid co-administration [7,8]. While cardiovascular risk upon enzalutamide use appears more modest, with most studies showing a 10–22% increased risk, it still warrants careful consideration—especially given that the target population often includes older men with pre-existing cardiovascular comorbidities [8,9]. Furthermore, evidence from Taiwan indicates that both abiraterone and enzalutamide are associated with a higher incidence of major adverse cardiovascular events compared to the baseline risk in the general population [10]. The clinical importance of cardiovascular toxicity in patients with prostate cancer has been further highlighted by the development of a national consensus in Taiwan, specifically focused on the management of cardiovascular risk in this patient group [11]. Despite the well-documented cardiovascular risks associated with ARPI therapy, no validated prediction tools currently exist for this patient group. Existing cardiovascular risk models, developed for non-PCa populations, may underestimate risk in ARPI-treated PCa patients [12,13]. This gap highlights the need for the development of specialized algorithms that can improve clinical decision-making and personalized cardiovascular monitoring.

Artificial intelligence (AI)-driven approaches have become increasingly valuable in enhancing the accuracy of prediction and classification tasks within medical research [14]. Deep learning techniques, in particular, allow researchers to implement multi-scale network architectures combined with output fusion strategies to optimize system performance [15]. Advanced machine learning models, such as the random survival forest (RSF), are capable of handling high-dimensional datasets and capturing complex nonlinear relationships among variables. Recent advances in machine learning and the availability of large-scale real-world data present an opportunity to develop more sophisticated prediction tools that could account for multiple risk factors and their complex interactions. Using RSF analysis, incorporating clinically relevant variables such as patient demographics, ARPI type, ADT modality, comorbidities, prior cardiovascular events, and treatment history, we aim to develop and validate a predictive model for major adverse cardiovascular events (MACEs) in this population, addressing the current lack of ARPI-specific prediction tools.

## 2. Materials and Methods

The data that support the findings of this study are available from the corresponding author upon reasonable request.

### 2.1. Data Acquisition and Characteristics

This investigation utilized the National Health Insurance Research Database (NHIRD), a comprehensive repository of healthcare claims data originating from Taiwan’s universal National Health Insurance Program. Established in 1995, this single-payer system provides near-universal coverage, encompassing approximately 99.8% of Taiwan’s population. For this study, we accessed data from the NHIRD spanning from 2009 to 2022. The NHIRD has been extensively validated for its accuracy in documenting cardiovascular-related conditions. Previous studies have confirmed high levels of precision in the recording of acute myocardial infarction [16], ischemic stroke [17], and diabetes mellitus [18]. Moreover, Taiwan’s mandatory death registration system has been verified to maintain exceptional accuracy [19]. To ensure patient confidentiality, all data extracted from the NHIRD underwent a thorough de-identification process prior to analysis. The study protocol received approval from the Institutional Review Board of Chang Gung Memorial Hospital (IRB number: 202300882B0), adhering to ethical guidelines for medical research involving human subjects.

### 2.2. PCa Cohort and ARPIs

**Figure 1:**
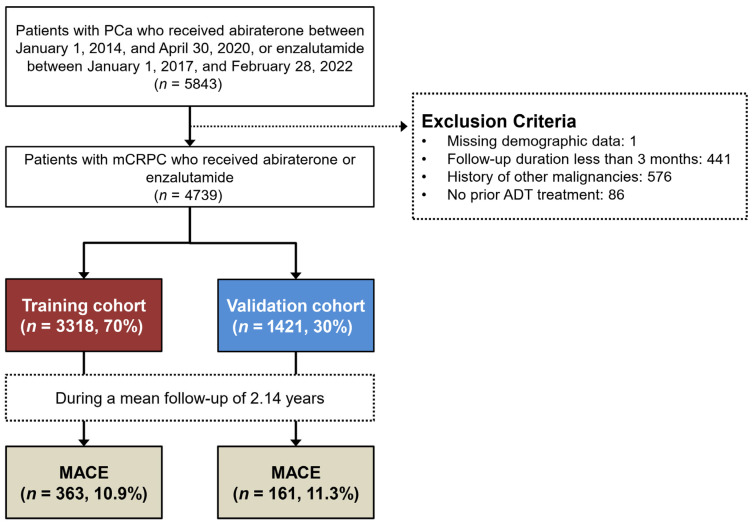
Study population selection flow diagram. PCa, prostate cancer; mCRPC, metastatic castration-resistant PCa; ADT, androgen deprivation therapy; MACE, major adverse cardiovascular event.

This study utilized the Taiwan Cancer Registry database, maintained by the Health and Welfare Data Science Center, to identify subjects with newly diagnosed PCa. Case identification was based on International Classification of Diseases (ICD) codes: ICD-9 code 185 and ICD-10 code C61. The cohort included patients who initiated treatment with androgen receptor signaling inhibitors (ARPIs), specifically abiraterone (Anatomical Therapeutic Chemical [ATC] classification system code L02BX03 or L02BX53) between 1 January 2014 and 30 April 2020 or enzalutamide (ATC code L02BB04) between 1 January 2017 and 28 February 2022. During the inclusion period, Taiwan’s National Health Insurance policy restricted reimbursement for abiraterone and enzalutamide to patients with confirmed metastatic castration-resistant PCa (mCRPC). In Taiwan, National Health Insurance mandates concurrent corticosteroid use (typically prednisone 5 mg twice daily) for abiraterone reimbursement, resulting in near-universal co-administration of corticosteroid with abiraterone in our study population. The index date was defined as the initiation of either ARPI. To facilitate model development and validation, the study population was randomly stratified into a training cohort (70%) and a validation cohort (30%). Subjects were excluded if they met any of the following conditions: incomplete demographic data, age below 40 years, follow-up duration less than 3 months, concurrent diagnosis of other malignancies, or no previous ADT treatment including orchiectomy (Figure 1).

### 2.3. Baseline Characteristics

The baseline characteristics included demographics (i.e., urbanization level, region, and age), previous docetaxel use, previous ADT treatment type (i.e., GnRH agonist, GnRH antagonist, and bilateral orchiectomy), comorbidities (outpatient or inpatient diagnoses), history of events (requiring hospitalization), and type of anti-androgen medications. The study protocol incorporated a comprehensive assessment of subjects’ medical history, utilizing standardized diagnostic codes from the ICD systems. Comorbidities were defined as conditions documented on or prior to the index date, establishing the baseline clinical profile for each participant. The spectrum of comorbidities evaluated encompassed a wide range of cardiovascular, metabolic, and systemic disorders. These included hypertensive disease, diabetes mellitus, coronary artery pathology, dyslipidemia, atrial dysrhythmia, peripheral vascular disease, chronic obstructive pulmonary syndrome, nephropathy (including renal replacement therapy), hepatic insufficiency, cardiac decompensation, myocardial ischemia, cerebrovascular events, and history of coronary revascularization procedures. In addition to comorbidities, the study protocol accounted for pharmacological interventions. Medication usage was operationally defined as the administration of specific therapeutic agents within a 90-day window preceding the designated index date, providing insight into the subjects’ pre-existing treatment regimens.

### 2.4. MACE and Follow-Up Protocol

The primary endpoint for the risk prediction model was the occurrence of a MACE, defined as a composite outcome encompassing HF hospitalization, myocardial infarction, ischemic stroke, and cardiovascular mortality. HF hospitalization was identified based on the primary admission diagnosis, a methodology previously validated in claims-based research [20]. Myocardial infarction and ischemic stroke were also determined by principal discharge diagnoses, which have also been validated [16,17]. Mortality data, including date, location, and causes of death, were extracted from the Taiwan Death Registry database, which has undergone rigorous validation [19]. Cardiovascular mortality was categorized to include fatalities attributable to acute myocardial infarction, sudden cardiac death, HF, stroke, cardiovascular procedures, and other cardiovascular etiologies [21]. The ICD codes employed for identifying baseline comorbidities and composite outcomes are detailed in Supplemental Table S1. Patients’ follow-up continued until the occurrence of a MACE, death, or the end of the database (31 December 2022), whichever occurred first.

### 2.5. Random Survival Forest Analysis

**Figure 2:**
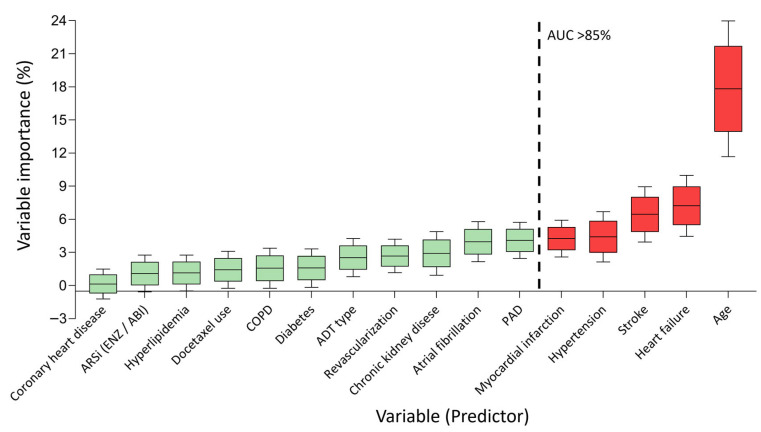
Variable importance distribution in random survival forest analysis. Distribution of variable importance across 16 parameters in the initial model. The five variables demonstrating the strongest correlation (AUC > 85%) were selected for the final model. The red bars correspond to five variables recognized as significant predictors in the final RSF model, whereas the green bars denote ten variables that were not deemed significant. Data derived from 1000 bootstrap samples. AUC, area under the curve.

Several machine learning approaches have been developed for analyzing survival (time-to-event) data, including models such as RSF and gradient-boosted survival models, as well as more recent deep learning methods like DeepSurv [22,23]. However, no single model performs best across all scenarios; thus, model selection should be guided by the characteristics of the data, the complexity of covariate–outcome relationships, and the primary analytic goal—whether it is prediction, interpretation, or causal inference. Among these approaches, RSF is widely regarded as a strong and interpretable nonparametric baseline for survival analysis, particularly in moderate-sized, structured clinical datasets [24]. Our study employed an RSF model to identify and evaluate predictors of MACEs. The model incorporated 16 clinically relevant variables routinely available during ARPI treatment (Table 1). These variables encompassed patients’ age, ARPI type, ADT modality, eight comorbidities (not including cardiovascular disease because its components have been included), four prior cardiovascular events requiring hospitalization, and previous docetaxel use. The RSF models were developed and conducted using R software version 4.3.2, utilizing the “randomForestSRC” package (Version: 3.4.1). The RSF analysis was initiated using the training cohort data. The model construction involved an iterative node-splitting process, commencing from the root node to create a binary survival tree. At each node, variables were randomly selected to determine optimal splits, maximizing survival disparities between daughter nodes. Trees were expanded to their maximum extent, ensuring each terminal node contained at least one distinct outcome. The cumulative hazard function was then estimated for each individual tree, and a final ensemble hazard function was obtained by averaging these estimates across the entire forest. In short, we constructed 500 trees with default ‘mtry’ = 500 and ‘nodesize’ = 15, using the log-rank splitting rule [25]. Variable importance (VIMP) was utilized to rank the prognostic capability of the 16 variables. Model performance was assessed using Harrell’s C-index. Following the development of the initial RSF model, a systematic reduction process was implemented to create a more clinically applicable model. The final model was required to maintain discriminatory performance comparable to more complex models (e.g., 5 vs. 16 features) while achieving satisfactory performance (area under the receiver operating characteristic curve [AUC] ≥85%). The refined RSF model incorporated the top five variables based on their VIMP scores (Figure 2). This approach balanced model complexity with clinical utility, facilitating practical implementation while maintaining robust predictive capabilities. To illustrate how the selected variable influences the risk of MACEs, a partial dependence plot was generated, accounting for the effects of all other covariates in the model. The generalizability of the model was evaluated using an external validation cohort, which consisted of a randomly selected 30% subset of the overall study population.

### 2.6. Predictive Behavior Analysis of RSF Variables

To elucidate the relationship between the risk of MACEs and each of the five selected variables, partial dependence plots were generated. The methodology for creating these plots adhered to protocols established in previous research [26]. The partial dependence analysis revealed a non-linear association between age and MACE risk. Specifically, the predicted survival rates for MACEs demonstrated a bimodal distribution, with decreased rates observed in both younger and older age cohorts. This observation prompted a clinically relevant dichotomization of the age variable: individuals aged < 65 or ≥75 years were grouped separately from those aged 65–74.9 years (Supplemental Figure S1A). The other four selected predictors were all binary variables (Supplemental Figure S1B–E).

### 2.7. Statistical Analysis

Comparative analyses of demographic characteristics and comorbidities between the training and validation cohorts were conducted using a range of statistical tests. Categorical variables were analyzed using chi-square tests, while continuous variables were assessed using independent-sample *t*-tests. The predictive performance of the models was evaluated using the area under the curve (AUC) metric. AUCs were computed and compared for both the initial 16-variable RSF model and the refined 5-variable RSF model. The final 5-variable RSF model underwent external validation using data from the validation cohort. To investigate the prognostic value of the identified predictors, the study population was stratified into four groups based on the number of applicable predictors (0–1, 2, 3, or ≥4). The risk of MACE of patients with different numbers of applicable predictors was compared using univariate Cox proportional hazards models, with the lowest risk group (0–1 features) serving as the reference category. The RSF models were developed and conducted using R software version 4.3.2, utilizing the “randomForestSRC” package. The RSF analysis was reiterated using the refined training cohort to ensure robustness. All other statistical analyses were conducted using SAS version 9.4 (SAS Institute, Cary, NC, USA).

## 3. Results

### 3.1. Patient Demographics and Clinical Outcomes

This investigation encompassed a cohort of 4739 PCa patients (Figure 1). The study population had a mean age of 75.1 ± 9.3 years at baseline. Regarding ARPI therapy, 2341 patients (49.4%) were administered abiraterone, while 2398 (50.6%) received enzalutamide. Prevalent comorbidities included hypertension (*n* = 2530, 53.4%), diabetes mellitus (*n* = 1338, 28.2%), and hyperlipidemia (*n* = 1285, 27.1%) (Table 1). The temporally split validation cohort, comprising 30% of the total sample, showed no significant differences from the training cohort in terms of baseline characteristics, including ARPI types and comorbidities, except for a minor geographic difference (*p* = 0.041).

Over a mean follow-up period of 2.1 years (standard deviation = 1.4 years), 524 individuals (11.1% of the cohort) experienced a MACE (Table 2). The specific MACE components were distributed as follows: ischemic stroke (*n* = 52, 1.1%), myocardial infarction (*n* = 44, 0.9%), heart failure hospitalization (*n* = 54, 1.1%), and cardiovascular death (*n* = 425, 9.0%). At the end of the follow-up period, the cumulative MACE rate was 11.1%. Notably, the all-cause mortality rate reached 67.8%, with PCa-specific mortality accounting for 60.4% of deaths (Table 2). A comprehensive comparison of baseline characteristics between patients who did and did not experience a MACE is presented in Supplemental Table S3. In addition, the information about other medications was detailed in Supplemental Table S2.

### 3.2. RSF Model Development and Predictor Selection

**Figure 3:**
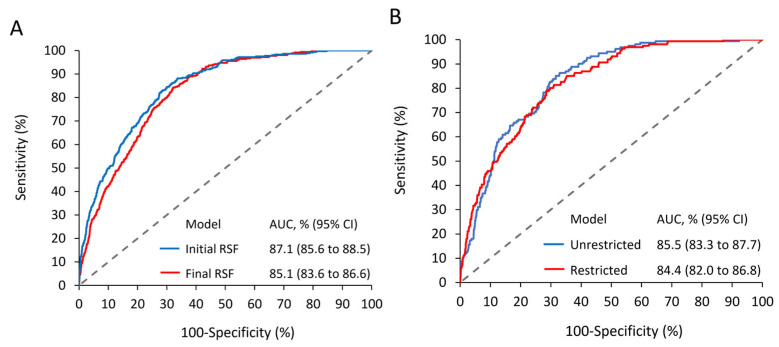
Random Survival Forest Model Performance. (**A**) Comparative analysis of the comprehensive 16-variable model versus optimized 5-variable model in derivation cohort. (**B**) Unrestricted and restricted AUC estimates of the final model in validation cohort. RSF, random survival forest; AUC, area under the curve.

The study cohort of 4739 patients was stratified into a training cohort (*n* = 3318, 70%) and a validation cohort (*n* = 1421, 30%). The initial RSF model incorporated all 16 variables from the training cohort (Figure 2). VIMP analysis, detailed in Supplemental Table S4, identified age as the most influential predictor (VIMP: 17.82%), followed by heart failure (VIMP: 7.22%) and stroke (VIMP: 6.45%). The performance metrics of RSF models with varying predictor numbers are presented in Supplemental Table S5. The comprehensive 16-variable RSF model demonstrated robust performance with an AUC of 87.1% (95% CI: 85.6–88.5%) (Figure 3A). A refined 5-variable model, retaining 85.1% AUC, was developed based on VIMP rankings, showcasing only a marginal 2% reduction in predictive power compared to the full model (Figure 3A). The final risk model incorporated the five most salient predictors: age, heart failure, stroke, hypertension, and myocardial infarction. External validation of this model yielded AUCs of 85.5% and 84.4% for unrestricted and restricted estimates, respectively, confirming its potential generalizability (Figure 3B). Partial dependency plots for the selected predictors are illustrated in Supplemental Figure S1. High-risk factors for MACEs were identified as age < 65 or ≥75 years, heart failure, stroke, hypertension, and myocardial infarction. These thresholds were verified through a Kaplan–Meier survival analysis using the training cohort data (Supplemental Figure S1).

### 3.3. Prognostic Significance of Identified Risk Factors

**Figure 4:**
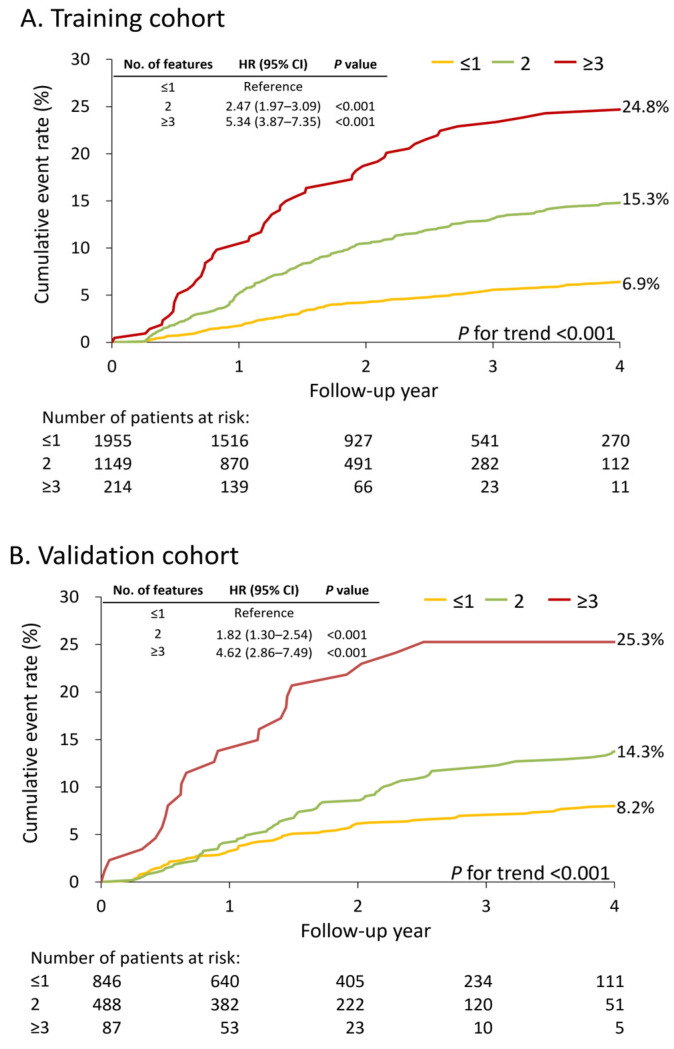
**Risk stratification based on predictor variables.** Cumulative MACE incidence stratified by predictor count in: (**A**) training and (**B**) validation cohorts. Number of features indicates presence of identified risk factors. MACE, major adverse cardiovascular event.

**Figure 5:**
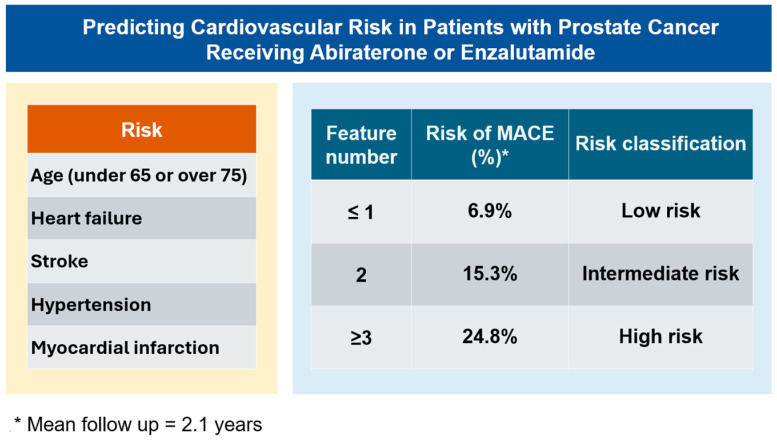
Risk stratification model for predicting cardiovascular events in patients with prostate cancer receiving abiraterone or enzalutamide. The **left** panel displays the five clinical risk factors included in the prediction model: age (categorized as under 65 or over 75 years), heart failure, stroke, hypertension, and myocardial infarction. The **right** panel presents the risk stratification system based on the number of risk factors present, with corresponding probabilities of major adverse cardiovascular events (MACE) and risk classifications.

Analysis of the cumulative incidence of MACEs revealed a positive correlation between event rates and the number of predictors present, consistent across both cohorts (Figure 4). In the training cohort, stratification by risk factor count yielded the following 4-year MACE rates: 6.9% for patients with 0–1 risk factors, 15.3% for those with 2 risk factors, and 24.8% for individuals with ≥3 risk factors (*p* trend < 0.001; Figure 4A). The validation cohort demonstrated similar trends, with 4-year MACE rates of 8.2%, 14.3%, and 25.3% for patients with 0–1, 2, and ≥3 risk factors, respectively (*p* trend < 0.001; Figure 4B). This replication of findings in the validation cohort substantiates the potential robustness of the risk stratification model. The risk stratification model for predicting MACEs in patients with PCa receiving abiraterone or enzalutamide is shown in Figure 5.

### 3.4. Sensitivity Analysis

To evaluate the robustness of our findings, we conducted a sensitivity analysis using an alternative RSF model that incorporated non-cardiovascular death as a competing risk (Supplemental Tables S6 and S7). The results were generally consistent with those of the primary Cox-based RSF model. When using a 5-variable model, the discrimination performance was slightly reduced but remained robust, with an AUC of 81.0% (95% CI: 79.1–82.9%). Three variables—age, heart failure, and myocardial infarction—were shared between the top five predictors in both models. Notably, the AUC declined from 80.3% to 79.7% when increasing the number of predictors from three to four, suggesting that the 3-variable model may be optimal in the competing risk-based framework. Importantly, all three of the top predictors identified in the competing risk-based RSF model were also among the top five variables in the Cox-based RSF model, indicating a high level of consistency between the two approaches. These findings support the robustness and generalizability of key predictors across different modeling frameworks.

## 4. Discussion

This investigation leveraged advanced machine learning techniques to establish a predictive framework for MACEs in patients with mCRPC receiving ARPIs (abiraterone/enzalutamide). Through RSF methodology, we identified five prognostic indicators: age, heart failure, stroke, hypertensive disease, and prior myocardial infarction. A notable finding was the stepwise elevation in MACE occurrence correlating with increasing risk factor burden. The model demonstrated remarkable predictive accuracy, achieving an AUC of 85.1% in initial testing and maintaining robust discriminatory capability (AUC: 85.5%) during external validation. Significantly, all identified predictors represent standard clinical parameters routinely documented in patient care. This risk stratification algorithm can easily be incorporated within electronic health record systems, facilitating automated cardiovascular risk assessment in real-time clinical practice for PCa patients undergoing ARPI therapy.

Our study provides novel evidence identifying five critical baseline predictors of MACEs in PCa patients receiving ARPIs: age, heart failure, stroke, hypertension, and myocardial infarction. Some potential physiological mechanisms may explain the association between the risk factors and elevated MACEs [27]. Our findings extend this observation, particularly highlighting the vulnerability of patients with pre-existing atherosclerotic cardiovascular conditions, such as stroke or myocardial infarction, when receiving ARPIs. Of note, abiraterone’s CYP17 inhibition achieves complete androgen blockade while triggering mineralocorticoid excess, leading to hypertension and fluid retention [28]. On the other hand, enzalutamide’s direct androgen receptor antagonism, while avoiding mineralocorticoid activation, may still induce hypertension and endothelial dysfunction [29]. Therefore, the potential impact of elevated blood pressure, fluid retention, and endothelial dysfunction effect by ARPI may further lead to hemodynamic stress, precipitate myocardial ischemia and coronary plaque instability, resulting in HF hospitalization and cardiovascular events. These pathophysiological changes appear particularly pronounced in patients with pre-existing cardiovascular conditions, explaining their increased susceptibility to adverse cardiovascular outcomes during ARPI therapy.

Our analysis revealed a distinctive U-shaped correlation between age and MACE risk in PCa patients, with elevated risks observed in both elderly (≥75 years) and younger (<65 years) populations. While advanced age is a well-established cardiovascular risk factor, explaining the increased MACE susceptibility in the elderly cohort receiving ARPI therapy, the mechanisms underlying elevated cardiovascular risk in younger patients remain less clear. Notably, early-onset PCa demonstrates a higher prevalence of inherited cancer predisposition genes, characterized by aggressive tumor behavior and poor outcomes [30]. Although our study found an association between younger age at prostate cancer diagnosis and increased cardiovascular risk, the underlying mechanisms remain uncertain. This relationship may be influenced by a range of factors, including lifestyle behaviors, baseline comorbidities, differential treatment exposures, or other unmeasured variables. Previous studies showed that some genetic alterations, including BRCA1, BRCA2, and ATM mutations, not only predispose to early-onset PCa but also correlate with increased cardiovascular risk and heart failure development [31,32]. Further mechanistic studies are needed to better understand this association and determine whether specific risk stratification or preventive strategies should be considered for younger prostate cancer patients.

Recent cardiovascular risk assessment guidelines from major oncology societies, including the European Society of Medical Oncology (ESMO) and American Society of Clinical Oncology (ASCO), emphasize the importance of pre-treatment cardiovascular evaluation for potentially cardiotoxic cancer therapies [33,34]. The Heart Failure Association of the European Society of Cardiology, collaborating with the International Cardio-Oncology Society, has proposed specific cardiovascular risk assessment protocols for androgen deprivation therapy, including abiraterone and enzalutamide [35]. While current recommendations suggest using established cardiovascular risk calculators to estimate 10-year atherosclerotic event risk, these tools were not specifically developed for PCa patients receiving androgen deprivation therapy. Our study addresses this critical gap by developing and validating the first multivariate predictive model specifically designed for this population. The MACE risk prediction model presented here offers enhanced clinical applicability for real-world cardiovascular risk assessment in PCa patients undergoing ARPI.

This investigation derives its robustness from development within a large-scale, longitudinal, population-representative cohort. The Taiwan NHIRD provides exceptional accuracy and completeness in documenting clinical diagnoses and medication prescriptions, effectively minimizing selection and recall bias, thus providing an optimal foundation for developing a reliable prediction model [36]. Our study uniquely applies machine learning methodology, specifically RSF, to identify risk factors for MACEs in PCa patients receiving ARPIs. Unlike traditional binary classification approaches, RSF employs a nonparametric ensemble method specifically designed for analyzing right-censored survival data [37,38]. This methodology enhances the traditional random forest approach by accommodating time-to-event analysis [39]. The model’s reliability was confirmed through comprehensive internal and pseudo-external validation procedures across diverse patient populations. Notably, the predictor variables comprise readily available patient demographics and clinical parameters, offering a practical and reliable approach for cardiovascular risk assessment prior to initiating ARPI therapy. Our study provides a solid foundation for the development of more advanced machine learning algorithms, as well as the integration of additional biomarkers to improve cardiovascular risk prediction in PCa patients. This groundwork not only supports future methodological innovation but also facilitates informed clinical decision-making and guides subsequent cardiovascular management strategies.

### Study Limitations

This study has several limitations. First, our analysis was constrained by the inherent limitations of the National Health Insurance Research Database, which lacks certain potential cardiovascular risk indicators, including blood pressure measurements, metabolic parameters (serum glucose, lipid profiles), and hormonal levels (FSH, luteinizing hormone, testosterone). The incorporation of these additional parameters could potentially enhance the model’s predictive accuracy. Second, the absence of data on smoking status, systolic blood pressure, total cholesterol, and high-density lipoprotein cholesterol prevented us from applying the SCORE2 or SCORE2-OP cardiovascular risk algorithms to our study population [35]. Third, as the risk prediction model was developed and validated using a Taiwanese cohort, its generalizability to other ethnic and racial populations remains to be established. This is particularly relevant given the well-documented disparities in cardiovascular risk profiles among Black and White patients with mCRPC. As such, external validation in multi-ethnic cohorts will be essential to confirm the model’s broader applicability. In addition, recalibration may be necessary when applying the model to populations with different baseline risks, healthcare systems, or treatment patterns.

## 5. Conclusions

This nationwide cohort investigation provided a validated predictive framework for MACEs in PCa patients receiving ARPIs, including abiraterone or enzalutamide. Through RSF methodology, we identified five critical clinical parameters with significant prognostic value: patient age, heart failure, prior stroke, hypertensive disease, and myocardial infarction. The resulting risk stratification model provides clinicians with an evidence-based tool for individualized cardiovascular risk assessment prior to initiating ARPI therapy. This approach enables more informed therapeutic decision-making and facilitates the development of personalized cardiovascular monitoring strategies in this vulnerable patient population.

## Figures and Tables

**Table 1:** The baseline characteristics of patients in the training and validation cohorts.

Variable	Total (*n* = 4739)	Training (*n* = 3318)	Validation (*n* = 1421)	*p* Value
Urbanization level of the residence				0.343
Low	748 (15.8)	513 (15.5)	235 (16.5)	
Moderate	1667 (35.2)	1182 (35.6)	485 (34.1)	
High	1282 (27.1)	880 (26.5)	402 (28.3)	
Very High	1042 (22.0)	743 (22.4)	299 (21.0)	
Region				0.041
North	2000 (42.2)	1445 (43.6)	555 (39.1)	
West	1220 (25.7)	833 (25.1)	387 (27.2)	
South	1390 (29.3)	951 (28.7)	439 (30.9)	
East	129 (2.7)	89 (2.7)	40 (2.8)	
Age, year	75.1 ± 9.3	75.0 ± 9.2	75.3 ± 9.6	0.380
Age group, year				0.108
40–49	21 (0.4)	13 (0.4)	8 (0.6)	
50–59	234 (4.9)	163 (4.9)	71 (5.0)	
60–69	1209 (25.5)	838 (25.3)	371 (26.1)	
70–79	1758 (37.1)	1270 (38.3)	488 (34.3)	
≥80	1517 (32.0)	1034 (31.2)	483 (34.0)	
Age group, year				0.253
<65	1245 (26.3)	860 (25.9)	385 (27.1)	
65–74	1531 (32.3)	1112 (33.5)	419 (29.5)	
≥75	1963 (41.4)	1346 (40.6)	617 (43.4)	
ARPI at index date				0.408
Abiraterone	2341 (49.4)	1626 (49.0)	715 (50.3)	
Enzalutamide	2398 (50.6)	1692 (51.0)	706 (49.7)	
Previous docetaxel use	1749 (36.9)	1229 (37.0)	520 (36.6)	0.770
ADT type before the index date				0.492
GnRH agonist	3886 (82.0)	2735 (82.4)	1151 (81.0)	
GnRH antagonist (Degarelix)	557 (11.8)	382 (11.5)	175 (12.3)	
Bilateral orchiectomy	296 (6.3)	201 (6.1)	95 (6.7)	
Comorbidities				
Hypertension	2530 (53.4)	1783 (53.7)	747 (52.6)	0.460
Diabetes mellitus	1338 (28.2)	936 (28.2)	402 (28.3)	0.955
Coronary heart disease	295 (6.2)	208 (6.3)	87 (6.1)	0.848
Hyperlipidemia	1285 (27.1)	906 (27.3)	379 (26.7)	0.653
Atrial fibrillation	180 (3.8)	124 (3.7)	56 (3.9)	0.737
Peripheral arterial disease	162 (3.4)	117 (3.5)	45 (3.2)	0.533
Chronic obstructive pulmonary disease	444 (9.4)	301 (9.1)	143 (10.1)	0.283
Chronic kidney disease or dialysis	1047 (22.1)	740 (22.3)	307 (21.6)	0.596
Cardiovascular disease *	809 (17.1)	569 (17.2)	240 (16.9)	0.828
History of event				
Myocardial infarction	94 (2.0)	68 (2.1)	26 (1.8)	0.619
Coronary revascularization	224 (4.7)	165 (5.0)	59 (4.2)	0.222
Heart failure	205 (4.3)	148 (4.5)	57 (4.0)	0.486
Stroke	300 (6.3)	206 (6.2)	94 (6.6)	0.599
Duration between PCa diagnosis and index, month	53.1 ± 45.0	52.5 ± 44.7	54.4 ± 45.7	0.193
Anti-androgen medication				
Flutamide	161 (3.4)	111 (3.4)	50 (3.5)	0.763
Bicalutamide	1824 (38.5)	1254 (37.8)	570 (40.1)	0.133
Cyproterone	366 (7.7)	270 (8.1)	96 (6.8)	0.103
Follow-up year	2.1 ± 1.4	2.1 ± 1.4	2.2 ± 1.4	0.756

Abbreviations: ADT, androgen deprivation therapy; GnRH, gonadotropin-releasing hormone; PCa, prostate cancer; ARPI, androgen receptor signaling inhibitors. * Any one of the following: coronary heart disease, peripheral arterial disease, myocardial infarction, or stroke; data are presented as frequency (percentage), mean ± standard deviation, or median [25th percentile, 75th percentile].

**Table 2:** The number of events at the end of follow-up in the training and validation cohorts.

Outcome	Total (*n* = 4739)	Training (*n* = 3318)	Validation (*n* = 1421)	*p* Value
**Cardiovascular event**				
Ischemic stroke	52 (1.1)	34 (1.0)	18 (1.3)	0.464
Myocardial infarction	44 (0.9)	33 (1.0)	11 (0.8)	0.468
Cardiovascular death	425 (9.0)	295 (8.9)	130 (9.2)	0.776
HF hospitalization	54 (1.1)	34 (1.0)	20 (1.4)	0.255
MACE *	524 (11.1)	363 (10.9)	161 (11.3)	0.695
**Other outcome**				
All-cause death	3211 (67.8)	2237 (67.4)	974 (68.5)	0.449
PCa related death	2863 (60.4)	1989 (60.0)	874 (61.5)	0.314

Abbreviations: HF, heart failure; MACE, major adverse cardiac events; PCa, prostate cancer. * Any one of the following: ischemic stroke, myocardial infarction, heart failure hospitalization, or cardiovascular death. Data are presented as frequencies (percentages).

## Data Availability

The data that support the findings of this study are available from the National Health Insurance Research Database (NHIRD), but restrictions apply to the availability of these data, which were used under license for the current study. Data are available from the corresponding author upon reasonable request and with permission of the National Health Insurance Administration.

## Supplementary Materials

The following supporting information can be downloaded at: https://www.mdpi.com/article/10.3390/cancers17152414/s1, Figure S1: Partial dependence analysis of final model predictors; Table S1: ICD diagnostic codes used in the study; Table S2: The concomitant medications of patients in the training and validation cohorts; Table S3: Baseline demographics of patients with or without developing subsequent MACE in the whole cohort; Table S4: VIMP and minimal depth of the initial RSF model in the training cohort; Table S5: Performance of RSF models with different numbers of predictors according to the rankings of the initial RSF model; Table S6: VIMP of the initial RSF model in the whole cohort, considering non-cardiovascular death a competing risk; Table S7: Performance of RSF models with different numbers of predictors according to the rankings of the initial RSF model, considering non-cardiovascular death a competing risk.

## Abbreviations

ADT	androgen deprivation therapy
ARPI	androgen receptor pathway inhibitor
GnRH	gonadotropin-releasing hormone
HF	heart failure
MACE	major adverse cardiovascular event
mCRPC	metastatic castration-resistant prostate cancer
NHIRD	National Health Insurance Research Database
PCa	prostate cancer
RSF	random survival forest
VIMP	variable importance
