# Supplementary material for: Predicting Cardiovascular Risk in Patients with Prostate Cancer Receiving Abiraterone or Enzalutamide by Using Machine Learning
Source: Cancers (Basel). 2025 Jul 22;17(15):2414. doi: 10.3390/cancers17152414 (PMC12345912; doi:10.3390/cancers17152414)
Supplement: Supplementary file 1 [file cancers-17-02414-s001.zip › cancers-3679140-supplementary.pdf]

Supplemental Materials

Title: Predicting Cardiovascular Risk in Patients with Prostate Cancer Receiving Abiraterone or Enzalutamide by Using Machine Learning

Contents

Supplemental Tables …………………………………...…………………….….... 2- 8

Table S1 ………………………………………………………………………….. 2-3

Table S2 ……………………………………………………………………….……..4

Table S3 …………………………………………………………………….….…… 5-7

Table S4 ……………………………………………………………………..………… 8

Table S5 ……………………………………………………………………..………… 9

Table S6 ……………………………………………………………………..…………10

Table S7 ……………………………………………………………………..…………11

Supplemental Figures……………………………………………………….............. 10-11

Figure S1 ……………………………………………………………………..……..…10-11

Supplemental Table S1. ICD diagnostic codes used in the study

Disease	ICD-9	ICD-10
Hypertension	401.x-405.x	I10.x-I15.x, N26.2x
Diabetes mellitus	250.x	E08.x-E13.x
Coronary heart disease	410.x-414.x	I20.x-I24.x
Hyperlipidemia	272.x	E77.x, E78.0x-E78.6x, E88.1x, E75.3x, E75.5x, E88.2x, E75.6x, E78.9x, E75.21, E75.22, E75.24, E71.30, E78.79, E78.81, E78.89, E88.89, E78.70
Atrial fibrillation	427.3x	I48.x
Peripheral arterial disease	440.x, 441.x, 443.x, 444.0x, 444.8x, 447.8x, 447.9x, 093.0, 437.3, 444.22, 447.1, 557.1, 557.9, V434	I70.x, I71.x, I73.x, I75.x, I77.1x, I79.0x, I79.1x, I79.2x, I77.3x, I77.9x, I79.8x, K55.1x, K55.8x, K55.9x, Z95.8x, Z95.9x, I74.3x-I74.5x, I74.8x, I74.0x, I77.89x
Chronic obstructive pulmonary disease	491.x, 492.x, 496.x	J41.x-J44.x
Chronic kidney disease	580.x-589.x, 403.x-404.x, 016.0x, 095.4x, 236.9x, 250.4x, 274.1x, 442.1x, 447.3x, 440.1x, 572.4x, 642.1x, 646.2x, 753.1x, 283.11, 403.01, 404.02, 446.21	A18.11x, D59.3x, E10.2x, E11.2x, E13.2x, I12.x, I13.x, K76.7x, M10.3x, M31.0x, N00.x-N08.x, N14.x, N15.0x, N15.8x, N15.9x, N16.x, N17.1x, N17.2x, N18.x, N19.x, N20.0x, N25.x, N26.1x, N26.9x, N27.x, Q61.x
Myocardial infarction	410.x, 412.x	I21.x-I22.x
Heart failure hospitalization	428.x	I50.x

Disease	ICD-9	ICD-10
Stroke	430.x-437.x	I60.x-I62.x, I66.x, I65.1x, I65.0x, I65.8x, I65.9x, I63.6x, I63.8x, I63.9x, G45.0x, G45.8x, G45.1x, G45.2x, G46.0x, G46.1x, G46.2x, G45.9x, G45.4x, G46.3x, G46.4x, G46.5x, G46.6x, G46.7x, G46.8x, I67.0x, I67.1x, I67.2x, I67.4x-I67.9x, I68.0x, I68.2x, I68.8x
Ischemic stroke	433.x-437.x	I66.x, I65.1x, I65.0x, I65.8x, I65.9x, I63.6x, I63.8x, I63.9x, G45.0x, G45.8x, G45.1x, G45.2x, G46.0x, G46.1x, G46.2x, G45.9x, G45.4x, G46.3x, G46.4x, G46.5x, G46.6x, G46.7x, G46.8x, I67.0x, I67.1x, I67.2x, I67.4x, I67.5x, I67.6x, I67.7x, I67.9x, I68.0x, I68.2x, I68.8x
Myocardial infarction	410.x	I21.x
Cardiovascular death	390.x-459.x	I01.x, I02.0x, I05-I15.x, I20.x-I25.x, I27.x, I30.x, I31.x, I32.x, I33.x-I52.x, I60.x-I71.x

Abbreviation: ICD, International Classification of Diseases.

Supplemental Table S2. The concomitant medications of patients in the training and validation cohorts

Variable	Total (*n* = 4,739)	Training (*n* = 3,318)	Validation (*n* = 1,421)	*P* value
NSAIDs	2,739 (57.8)	1,913 (57.7)	826 (58.1)	0.763
Antiplatelet agents (Aspirin/ Clopidogrel/ Ticagrelor)	1,201 (25.3)	843 (25.4)	358 (25.2)	0.877
Cox-2 inhibitor	907 (19.1)	624 (18.8)	283 (19.9)	0.374
Anticoagulant agents (Warfarin/ NOACs)	229 (4.8)	163 (4.9)	66 (4.6)	0.694
Oral hypoglycemic agents	1,150 (24.3)	808 (24.4)	342 (24.1)	0.834
Insulin	190 (4.0)	128 (3.9)	62 (4.4)	0.417
ACEI / ARB / ARNI	1,509 (31.8)	1,044 (31.5)	465 (32.7)	0.394
Beta-blocker	929 (19.6)	641 (19.3)	288 (20.3)	0.451
Dihydropyridine calcium channel blockers	1,681 (35.5)	1,178 (35.5)	503 (35.4)	0.944
MRAs (Spironolactone/ Elephrone)	250 (5.3)	181 (5.5)	69 (4.9)	0.398
Loop diuretics	858 (18.1)	593 (17.9)	265 (18.7)	0.525
Thiazide	227 (4.8)	169 (5.1)	58 (4.1)	0.135
Statin	1,212 (25.6)	839 (25.3)	373 (26.3)	0.486
Fibrate	115 (2.4)	84 (2.5)	31 (2.2)	0.473

Abbreviation: NSAIDs, non-steroidal anti-inflammatory drugs; Cox-2, cyclooxygenase-2; NOAC, novel oral anticoagulants; ACEI, angiotensin-converting enzyme inhibitor; ARB, angiotensin receptor blocker; ARNI, angiotensin receptor-neprilysin inhibitor; MRAs, mineralocorticoid receptor antagonists;

Data are presented as frequency (percentage).

Supplemental Table S3. **Baseline demographics of patients with or without developing subsequent MACE in the whole cohort**

Variable	Total (*n* = 4,739)	MACE (*n* = 524)	Non-MACE (*n* = 4,215)	*P* value
Urbanization level				0.357
Low	748 (15.8)	95 (18.1)	653 (15.5)	
Moderate	1,667 (35.2)	171 (32.6)	1,496 (35.5)	
High	1,282 (27.1)	140 (26.7)	1,142 (27.1)	
Very High	1,042 (22.0)	118 (22.5)	924 (21.9)	
Region				0.820
North	2,000 (42.2)	222 (42.4)	1,778 (42.2)	
West	1,220 (25.7)	129 (24.6)	1,091 (25.9)	
South	1,390 (29.3)	156 (29.8)	1,234 (29.3)	
East	129 (2.7)	17 (3.2)	112 (2.7)	
Age, year	75.1 ± 9.3	78.3 ± 9.0	74.7 ± 9.3	<0.001
Age group				<0.001
40-49	21 (0.4)	2 (0.4)	19 (0.5)	
50-59	234 (4.9)	13 (2.5)	221 (5.2)	
60-69	1,209 (25.5)	84 (16.0)	1,125 (26.7)	
70-79	1,758 (37.1)	185 (35.3)	1,573 (37.3)	
≥80	1,517 (32.0)	240 (45.8)	1,277 (30.3)	
ADT type before the index date				0.380
GnRH agonist	3,886 (82.0)	441 (84.2)	3,445 (81.7)	
GnRH antagonist (Degarelix)	557 (11.8)	53 (10.1)	504 (12.0)	
Bilateral orchiectomy	296 (6.3)	30 (5.7)	266 (6.3)	
Comorbidities				

Variable	Total (*n* = 4,739)	MACE (*n* = 524)	Non-MACE (*n* = 4,215)	*P* value
Hypertension	2,530 (53.4)	349 (66.6)	2,181 (51.7)	<0.001
Diabetes mellitus	1,338 (28.2)	184 (35.1)	1,154 (27.4)	<0.001
Coronary heart disease	295 (6.2)	59 (11.3)	236 (5.6)	<0.001
Hyperlipidemia	1,285 (27.1)	172 (32.8)	1,113 (26.4)	0.002
Atrial fibrillation	180 (3.8)	37 (7.1)	143 (3.4)	<0.001
Peripheral arterial disease	162 (3.4)	25 (4.8)	137 (3.3)	0.071
Chronic obstructive pulmonary disease	444 (9.4)	70 (13.4)	374 (8.9)	0.001
Chronic kidney disease or dialysis	1,047 (22.1)	144 (27.5)	903 (21.4)	0.002
Cardiovascular disease*	809 (17.1)	141 (26.9)	668 (15.9)	<0.001
History of event				
Myocardial infarction	94 (2.0)	31 (5.9)	63 (1.5)	<0.001
Coronary revascularization	224 (4.7)	47 (9.0)	177 (4.2)	<0.001
Heart failure	205 (4.3)	57 (10.9)	148 (3.5)	<0.001
Stroke	300 (6.3)	49 (9.4)	251 (6.0)	0.003
Duration between PCa diagnosis and index, month	53.1 ± 45.0	58.9 ± 45.5	52.4 ± 44.9	0.002
Medication				
ARSI at index date				0.040
Abiraterone	2,341 (49.4)	281 (53.6)	2,060 (48.9)	
Enzalutamide	2,398 (50.6)	243 (46.4)	2,155 (51.1)	
Other type of anti-androgen				
Flutamide	161 (3.4)	20 (3.8)	141 (3.4)	0.574
Bicalutamide	1,824 (38.5)	248 (47.3)	1,576 (37.4)	<0.001
Cyproterone	366 (7.7)	35 (6.7)	331 (7.9)	0.343

Variable	Total (*n* = 4,739)	MACE (*n* = 524)	Non-MACE (*n* = 4,215)	*P* value
Previous docetaxel use	1,749 (36.9)	171 (32.6)	1,578 (37.4)	0.032
Concomitant medication				
NSAIDs	2,739 (57.8)	321 (61.3)	2,418 (57.4)	0.089
Antiplatelet agents (Aspirin/ Clopidogrel/ Ticagrelor)	1,201 (25.3)	198 (37.8)	1,003 (23.8)	<0.001
Cox-2 inhibitor	907 (19.1)	111 (21.2)	796 (18.9)	0.207
Anticoagulant agents (Warfarin/ NOACs)	229 (4.8)	39 (7.4)	190 (4.5)	0.003
Oral hypoglycemic agents	1,150 (24.3)	163 (31.1)	987 (23.4)	<0.001
Insulin	190 (4.0)	26 (5.0)	164 (3.9)	0.239
ACEI / ARB / ARNI	1,509 (31.8)	223 (42.6)	1,286 (30.5)	<0.001
Beta-blocker	929 (19.6)	141 (26.9)	788 (18.7)	<0.001
Dihydropyridine calcium channel blockers	1,681 (35.5)	242 (46.2)	1,439 (34.1)	<0.001
MRAs (Spironolactone/ Elephrone)	250 (5.3)	58 (11.1)	192 (4.6)	<0.001
Loop diuretics	858 (18.1)	126 (24.1)	732 (17.4)	<0.001
Thiazide	227 (4.8)	43 (8.2)	184 (4.4)	<0.001
Statin	1,212 (25.6)	168 (32.1)	1,044 (24.8)	<0.001
Fibrate	115 (2.4)	21 (4.0)	94 (2.2)	0.013
Follow up year	2.1 ± 1.4	1.9 ± 1.2	2.2 ± 1.4	<0.001

Abbreviation: MACE, major adverse cardiac events; ADT, androgen deprivation therapy; GnRH, gonadotropin releasing hormone; PCa, prostate cancer; ARSI, androgen receptor signaling inhibitors; NSAIDs, non-steroidal anti-inflammatory drugs; Cox-2, cyclooxygenase-2; NOAC, novel oral anticoagulants; ACEI, angiotensin-converting enzyme inhibitor; ARB, angiotensin receptor blocker; ARNI, angiotensin receptor-neprilysin inhibitor; MRAs, mineralocorticoid receptor antagonists;

* Anyone of coronary heart disease, peripheral arterial disease, myocardial infarction and stroke;

Data are presented as frequency (percentage), mean ± standard deviation or median [25th percentile, 75th percentile].

Supplemental Table S4. VIMP and minimal depth of the initial RSF model in the training cohort.

Features	VIMP (%)	Rank of VIMP	Minimum depth
1. Age	17.82	1	1.57
2. Heart failure	7.22	2	1.83
3. Stroke	6.45	3	3.02
4. Hypertension	4.42	4	2.67
5. Myocardial infarction	4.25	5	3.15
6. Peripheral arterial disease	4.09	6	4.57
7. Atrial fibrillation	3.97	7	3.23
8. Chronic kidney disease or dialysis	2.91	8	4.19
9. Coronary revascularization	2.68	9	3.45
10. ADT type before the index date	2.53	10	3.63
11. Diabetes mellitus	1.59	11	3.86
12. Chronic obstructive pulmonary disease	1.56	12	3.59
13. Previous docetaxel use	1.43	13	4.61
14. Hyperlipidemia	1.14	14	4.28
15. Anti-androgen medication at index date	1.09	15	4.01
16. Coronary heart disease	0.14	16	3.87

Abbreviation: VIMP, variable importance; RSF, random survival forest; ADT, androgen deprivation therapy

Supplemental Table S5. Performance of RSF models with different numbers of predictors according to the rankings of the initial RSF model

Feature numbers	AUC, % (95% CI)
Top 1 feature	83.8 (82.1, 85.4)
Top 2 features	84.8 (83.3, 86.3)
Top 3 features	84.9 (83.4, 86.4)
Top 4 features	84.5 (82.9, 86.0)
**Top 5 features***	**85.1 (83.6, 86.6)**
Top 6 features	85.3 (83.8, 86.8)
Top 7 features	84.9 (83.4, 86.4)
Top 8 features	85.1 (83.6, 86.6)
Top 9 features	84.8 (83.2, 86.4)
Top 10 features	85.8 (84.3, 87.3)
Top 11 features	86.0 (84.5, 87.5)
Top 12 features	86.4 (84.9, 87.9)
Top 13 features	86.3 (84.7, 87.8)
Top 14 features	86.8 (85.3, 88.2)
Top 15 features	87.0 (85.5, 88.4)
All (16 features)	87.1 (85.6, 88.5)

Abbreviations: RSF, random survival forest; AUC, area under curve; CI, confidence interval.

* The final selected model.

Supplemental Table S6. VIMP of the initial RSF model in the whole cohort, considering non-cardiovascular death a competing risk

Features	VIMP (%)	Rank of VIMP
1. Heart failure	18.05	1
2. Age	11.50	2
3. Myocardial infarction	9.36	3
4. Coronary revascularization	6.52	4
5. Peripheral arterial disease	6.21	5
6. Stroke	5.56	6
7. Anti-androgen medication at index date	5.35	7
8. Atrial fibrillation	4.37	8
9. Coronary heart disease	4.11	9
10. ADT type before the index date	3.58	10
11. Diabetes mellitus	2.47	11
12. Hypertension	2.46	12
13. Previous docetaxel use	2.40	13
14. Chronic kidney disease or dialysis	0.72	14
15. Chronic obstructive pulmonary disease	0.35	15
16. Hyperlipidemia	-0.20	16

Abbreviation: VIMP, variable importance; RSF, random survival forest; ADT, androgen deprivation therapy

Supplemental Table S7. Performance of RSF models with different numbers of predictors according to the rankings of the initial RSF model, considering non-cardiovascular death a competing risk

Feature numbers	AUC, % (95% CI)
Top 1 feature	55.4 (53.5 ,57.2)
Top 2 features	79.6 (77.6 ,81.6)
Top 3 features	80.3 (78.3 ,82.2)
Top 4 features	79.7 (77.6 ,81.7)
**Top 5 features**	**81.0 (79.1 ,82.9)**
Top 6 features	81.3 (79.5 ,83.2)
Top 7 features	82.8 (81.1 ,84.4)
Top 8 features	83.0 (81.3 ,84.7)
Top 9 features	83.1 (81.4 ,84.8)
Top 10 features	84.0 (82.3 ,85.7)
Top 11 features	84.8 (83.2 ,86.4)
Top 12 features	85.4 (83.8 ,86.9)
Top 13 features	85.6 (83.9 ,87.2)
Top 14 features	85.5 (83.8 ,87.2)
Top 15 features	86.1 (84.4 ,87.8)
All (16 features)	86.7 (85.1 ,88.4)

Abbreviations: RSF, random survival forest; AUC, area under curve; CI, confidence interval.

* The final selected model.

Supplemental Figure S1. Partial Dependence Analysis of Final Model Predictors. Visualization of predictive factor relationships in derivation cohort using partial dependence plots. RSF, random survival forest.

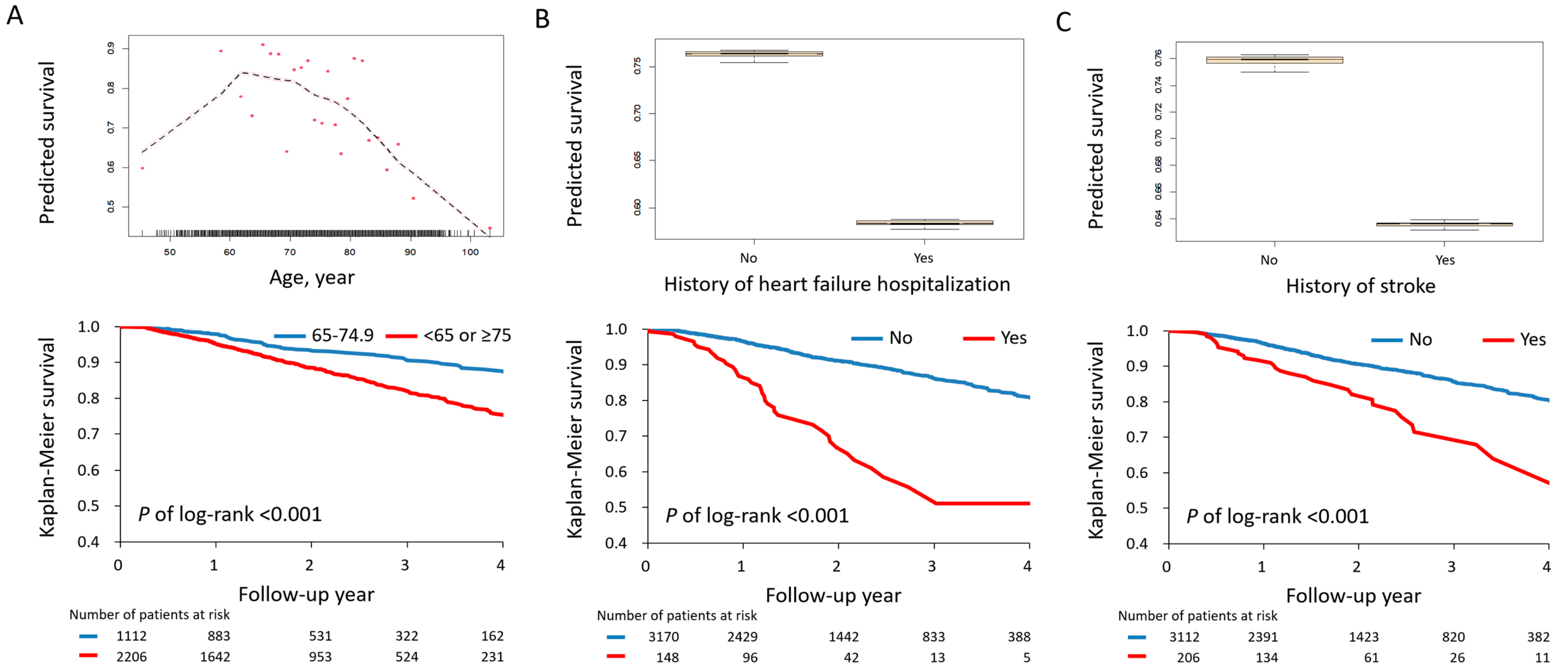

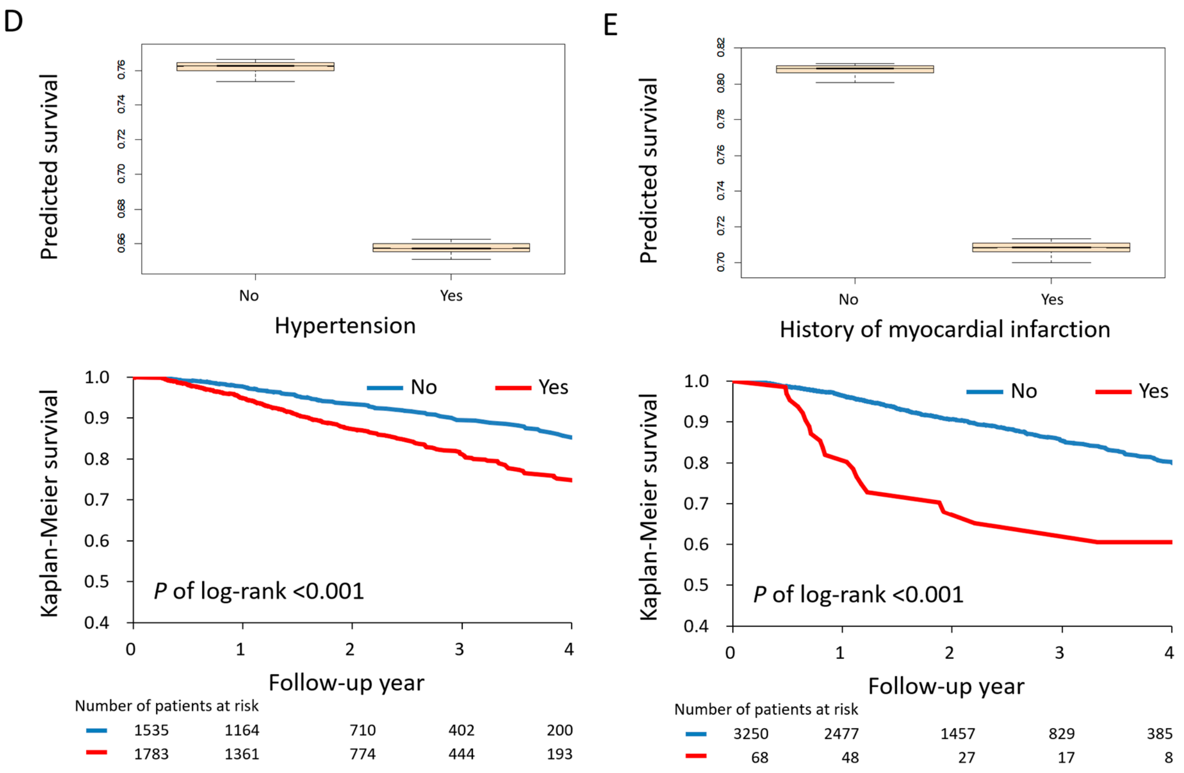
D
Predicted survival
0.66
0.68
0.70
0.72
0.74
0.76
No
Yes
Hypertension
Kaplan-Meier survival
1.0
0.9
0.8
0.7
0.6
0.5
0.4
No
Yes
P of log-rank <0.001
0
1
2
3
4
Follow-up year
Number of patients at risk
1535 1164 710 402 200
1783 1361 774 444 193
E
Predicted survival
0.70
0.72
0.74
0.76
0.78
0.80
0.82
No
Yes
History of myocardial infarction
Kaplan-Meier survival
1.0
0.9
0.8
0.7
0.6
0.5
0.4
No
Yes
P of log-rank <0.001
0
1
2
3
4
Follow-up year
Number of patients at risk
3250 2477 1457 829 385
68 48 27 17 8
